# Demography and disorders of English Cocker Spaniels under primary veterinary care in the UK

**DOI:** 10.1186/s40575-023-00128-x

**Published:** 2023-05-19

**Authors:** Karolina S. Engdahl, Dave C. Brodbelt, Carla Cameron, David B. Church, Åke Hedhammar, Dan G. O’Neill

**Affiliations:** 1 Department of Clinical Sciences, Swedish University of Agricultural Sciences, PO Box 7054, 750 07 Uppsala, Sweden; 2 Pathobiology and Population Science, The Royal Veterinary College, North Mymms, Hawkshead Lane, Hatfield, AL9 7TA Herts UK; 3 Clinical Science and Services, The Royal Veterinary College, North Mymms, Hawkshead Lane, Hatfield, AL9 7TA Herts UK

**Keywords:** VetCompass, Electronic patient record, EPR, Breed, Dog, Epidemiology, Primary-care, Veterinary, Pedigree, English Cocker Spaniel

## Abstract

**Background:**

The English Cocker Spaniel (ECS) is a common family dog in the UK. This study aimed to describe demography, morbidity, and mortality in ECS under primary veterinary care in the UK during 2016 using data from the VetCompass™ Programme. This study hypothesised that the prevalence of aggression is higher in male than female ECS, and higher in solid-coloured than bi-coloured ECS.

**Results:**

English Cocker Spaniels comprised 10,313/336,865 (3.06%) of dogs under primary veterinary care during 2016. The median age was 4.57 years (inter-quartile range (IQR) 2.25–8.01) and the median adult bodyweight was 15.05 kg (IQR 13.12–17.35). The annual proportional birth rate was relatively stable between 2.97–3.51% from 2005–2016. The most common specific diagnoses were periodontal disease (*n* = 486, prevalence 20.97%, 95% confidence interval (CI): 19.31–22.62), otitis externa (*n* = 234, 10.09%, 95% CI: 8.87–11.32), obesity (*n* = 229, 9.88%, 95% CI: 8.66–11.09), anal sac impaction (*n* = 187, 8.07%, 95% CI: 6.96–9.18), diarrhoea (*n* = 113, 4.87%, 95% CI: 4.00–5.75), and aggression (*n* = 93, 4.01%, 95% CI: 3.21–4.81). The prevalence of aggression was higher in males (4.95%) than in females (2.87%) (*P* = 0.015) and in solid-coloured (7.00%) than in bi-coloured dogs (3.66%) (*P* = 0.010). The median age at death was 11.44 years (IQR 9.46–13.47) and the most common grouped causes of death were neoplasia (*n* = 10, 9.26%, 95% CI: 3.79–14.73), mass-associated disorders (*n* = 9, 8.33%, 95% CI: 4.45–15.08), and collapse (*n* = 8, 7.41%, 95% CI: 3.80–13.94).

**Conclusions:**

Periodontal disease, otitis externa, and obesity are identified as the most common health issues for ECS, and neoplasia and mass-associated disorders as the most common reasons for death. The prevalence of aggression was higher in males and solid-coloured dogs. The results can aid veterinarians in giving evidence-based health and breed choice information to dog owners and highlights the importance of thorough oral examination and body condition score evaluation during routine veterinary examination of ECS.

## Abstract (plain language summary)

The English Cocker Spaniel (ECS) is a popular family dog in the UK, but there is limited information regarding common disorders affecting the breed. The goal of this study was to describe demography (age, sex, neuter, and bodyweight), disease occurrence, lifespan, and reasons for death in ECS by using data from the VetCompass™ Programme. The VetCompass™ Programme collects information from anonymised clinical records of dogs attending first-opinion veterinary practices in the UK. This study hypothesised that aggression is more common in males than in females, and in solid-coloured than in bi-coloured ECS dogs.

English Cocker Spaniels comprised 10,313/336,865 (3.06%) of dogs under primary veterinary care during 2016. Breed popularity did not vary much from 2005 to 2016, comprising around 3% of all dogs born each year. The average age of dogs in 2016 was 4.57 years and the average adult bodyweight was 15.05 kg. The most common disorders were periodontal disease (infection of the tissues that hold the teeth in place, affecting 20.97% of the dogs), inflammation of the external ear canal (10.09%), obesity (9.88%), anal sac impaction (8.07%), diarrhoea (4.87%), and aggression (4.01%). Aggression was more common in males (4.95%) than in females (2.87%) and in solid-coloured (7.00%) than in bi-coloured (3.66%) dogs. The frequency of aggression also varied across the four most common solid colours (black, liver, golden, red), with golden-coloured dogs showing the most aggression (12.08%). The average lifespan was 11.44 years and the most common cause of death was tumours.

This study shows that first-opinion clinical records can help us to understand and enhance breed health. The results can guide veterinarians in giving breed-adapted information to owners of ECS and help breeders to optimise breeding decisions. Further, this information can be used by future ECS owners to make more informed decisions when acquiring a dog if avoidance of aggression is a key priority. Periodontal disease was the most common condition affecting the breed, which highlights the importance of regular veterinary dental checks and as well as tooth brushing in ECS.

## Background

The English Cocker Spaniel (ECS) is a gundog first bred to hunt woodcock, giving the modern breed its name when the breed was formally recognised in 1893 [1]. Today the ECS is a popular family dog promoted as “the merry cocker” by the UK Kennel Club [1]. The breed was the 5^th^ most commonly microchipped breed in the UK between 2004 and 2014 and the second most common registered breed in the UK Kennel Club in 2016 but was just the 56^th^ most common breed registered in the American Kennel Club during 2016 [2–4]. The breed popularity in the UK has been consistent in recent years with between 21,663 to 23,927 dogs (9.32–10.26% of all registrations) registered by the UK Kennel Club annually from 2012 to 2019 [4]. The lifespan of the ECS is described as over 10 years by the UK Kennel Club, in concordance with two studies evaluating the longevity of dogs attending first-opinion veterinary practices and Kennel Club registered dogs in the UK, which reported median lifespans of 10.8 and 11.5 years, respectively [5, 6].

The ECS is currently classified as Breed Watch category 1 by the UK Kennel Club, meaning that there are no health issues of concern highlighted for special attention by judges [1, 7]. However, the UK Kennel Club’s Assured Breeders must screen their ECS for the ocular disorders primary glaucoma, progressive retinal atrophy, and retinal pigment epithelial dystrophy, as well as familial nephropathy, acral mutilation syndrome, and the ECS specific disorder adult onset neuropathy before breeding [1, 8]. The UK Kennel Club also recommends screening for hip dysplasia and has listed pancreatitis, immune-mediated haemolytic anaemia (IMHA), and thyroid conditions as current key priorities for ECS [1]. However, there is limited data on the frequency of many of these disorders despite their recommendation for pre-breeding assessment. A review of breed predisposition of diseases across all dog breeds identified 63 disorders with some evidence of predisposition for the ECS [9], including otitis externa [10], immune-mediated haemolytic anaemia [11, 12], pancreatitis [13], chronic hepatitis [14], and several ocular conditions such as glaucoma [15]. Further, ECS are over-represented in studies evaluating canine aggression [16, 17], and the prevalence of aggression has been generally reported as higher in males than in females [16, 18–20]. It has also been reported that solid-coloured ECS are more likely to show signs of aggression than bi-coloured or tri-coloured, and that golden and red-coated ECS are more likely to show aggression than black-coated [20–23]. However, the majority of studies reporting aggression in ECS were published over 15 years ago [16, 17, 19–21] and were based on study populations attending referral animal behavioural clinics [16, 19, 20], which limits the generalisability of the results to the current general ECS population.

It is important to differentiate between prevalence and predisposition when interpreting disorder occurrence and impact. Prevalence describes the proportion of affected individuals in a population while predisposition reflects the proportion of affected individuals within one group compared to another group, such as disorder risk in one breed compared to the overall dog population [24]. A disorder with high prevalence may still have a high impact on the overall health and welfare of a breed even without there being a breed predisposition. Conversely, a disease predisposition does not imply that the disorder is necessarily a high priority for that breed, because the prevalence and/or severity of a rare disorder may be low despite a breed predisposition [24]. Information regarding disorder prevalence as well as the severity and duration of the disorder is necessary to fully assess the welfare impact at the population level [25, 26], and the current study adds to our ability to assess the welfare impact for ECS by reporting the prevalence of common disorders.

Demographic data, such as breed, sex, and neuter status, as well as information on disease occurrence, are routinely collected in veterinary primary-care practice management systems [27]. Using primary-care data in research benefits from the inclusion of all dogs under veterinary care and all disorders recorded in the electronic patient record (EPR), and a high generalisability of the results to the wider dog population as over 75% of the wider national dog population is registered for primary veterinary care in the UK [27, 28]. Using veterinary clinical data from the VetCompass™ Programme [29], this study aimed to characterise the demography, common disorders, and longevity of the general population of ECS under primary veterinary care in the UK during 2016. Based on prior evidence, this study hypothesised that the prevalence of aggression is higher in males than in females [16, 18–20] and that the prevalence varies with coat colour with a higher prevalence in solid-coloured dogs [21, 22]. The results from the current study could provide a reliable framework to assist reforms in breeding practices and ultimately contribute to improved health and welfare of ECS.

## Materials and methods

The study population included all dogs under primary veterinary care at clinics participating in the VetCompass™ Programme during 2016. Dogs under veterinary care were defined as those with either a) at least one EPR (VeNom diagnosis term, free-text clinical note, treatment, or bodyweight) recorded during 2016 or b) at least one EPR recorded both before 2016 and during 2017. The VetCompass™ Programme collates de-identified EPR data from primary-care veterinary practices in the UK for epidemiological research [29]. Data fields available for VetCompass™ researchers include a unique animal identifier from each practice management system provider along with species, breed, date of birth, colour, sex, neuter status, and bodyweight, and clinical information from free-form text clinical notes, summary diagnosis terms (VeNom codes), and treatment with relevant dates. Ethics approval was obtained from the RVC Ethics and Welfare Committee (reference number 2015/1369).

Dogs recorded as just Cocker Spaniels or ECS in the EPRs were categorised as ECS while all remaining variants of Cocker Spaniels including American Cocker Spaniels and Cocker Spaniel crossbreeds were categorised along with all other types of dogs as non-ECS. The bodyweight, sex, neuter status, and age for ECS under veterinary care during 2016 were described. *All-age Bodyweight* (Kg) described all available bodyweight and date combinations. *Adult Bodyweight* (Kg) described the mean bodyweight recorded from all bodyweight data for dogs aged over 18 months and was categorised into 6 groups (< 12.0, 12.0 to < 14.0, 14.0 to < 16.0, 16.0 to < 18.0, 18.0 to < 20.0, ≥ 20.0). *Neuter* described the status of the dog (entire or neutered) at the final EPR. *Age* (years) described the age at December 31^st^, 2016 and was categorised into 9 groups (< 1.0, 1.0 to < 2.0, 2.0 to < 3.0, 3.0 to < 5.0, 5.0 to < 7.0, 7.0 to < 9.0, 9.0 to < 11.0, 11.0 to < 13.0, ≥ 13.0).

A cross-sectional analysis of cohort clinical data of ECS registered at participating practices was used to estimate the one-year period prevalence of the most commonly diagnosed disorders. Sample size calculations estimated that 1,861 dogs would need to be sampled from a population of 10,313 dogs to report a disorder with a 1.50% expected prevalence, 95% confidence level, and a 0.50% margin of error [30]. Clinical records were manually reviewed in detail in a randomly selected subset of dogs to extract the most definitive diagnoses recorded for all disorders that existed during 2016 and to manually link this to the most appropriate VeNom term as previously described [31]. The extracted diagnosis terms were mapped to a dual hierarchy of precision for analysis: fine-level precision and grouped-level precision [31]. Briefly, fine-level precision terms described the original extracted terms at the maximal diagnostic precision recorded within the clinical notes (e.g. *inflammatory bowel disease* would remain as *inflammatory bowel disease*). Disorders described within the clinical notes using presenting sign terms (e.g. ‘vomiting’ or 'vomiting and diarrhoea'), but without a formal clinical diagnostic term being recorded, were included using the first sign listed (e.g. vomiting). Grouped-level precision terms mapped the original diagnosis terms to a general level of diagnostic precision (e.g. *inflammatory bowel disease* would map to *enteropathy*). Elective (e.g. neutering) or prophylactic (e.g. vaccination) clinical events were not included. No distinction was made between pre-existing and incident disorder presentations. Mortality data (recorded cause, date, and method of death) in this subset of dogs were extracted on all deaths at any date during the available EPR data.

Following data checking for internal validity and cleaning in Excel (Microsoft Office Excel 2013, Microsoft Corp.), analyses were conducted using R version 4.0.0 [32]. Annual proportional birth rates described the relative proportion of ECS compared with all dogs that were born in each year from 2005–2016 from the cohort under veterinary care in 2016. The figure illustrating annual proportional birth rates was generated with the R package ggplot2 [33]. All bodyweight data with their associated dates at any age of dog were used to generate individual bodyweight growth curves for male and female ECS by plotting age-specific bodyweights overlaid with a cross medians line using the R package ggplot2 [33].

One-year (2016) period prevalence values were reported along with 95% confidence intervals (CI) that described the probability of diagnosis at least once during 2016. The CI estimates were derived from standard errors based on an approximation to the normal distribution (Wald CI) for disorders with ten or more events [34] or the Wilson approximation method for disorders with fewer than ten events [35], using the binom.approx() and binom.wilson() functions from the R-package epitools [36]. Prevalence values were reported overall and separately for males and females. Coat colour data were retrieved and associations between coat colour and aggression were evaluated. Median age (years) as defined above was reported for each of the most common diagnoses at the fine-level and group-level. The 10 most common disorders at group-level precision in each of three age bands (< 2 years, 2–7 years, and > 7 years) were identified and the prevalence of each of these disorders through life up to the age of 14 is presented using loess curves in a figure generated with the R packages ggplot2, cowplot, and ggpubr [33, 37, 38]. A combination of the Shapiro–Wilk test and visual assessment of histograms was used to assess the normality of continuous variables. The two-proportion z-test was used to compare proportions, the chi-square test to compare categorical variables, and the Mann–Whitney U test to compare continuous variables as these deviated from normality [34]. Statistical significance was set at the 5% level.

## Results

### Demography

**Fig. 1:**
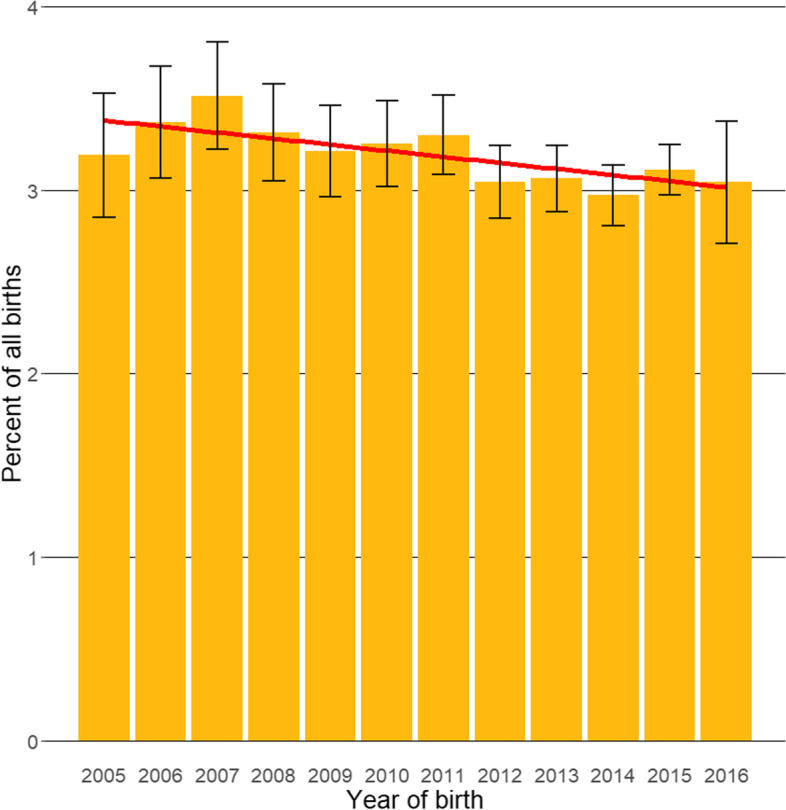
Annual proportional birth rates (2005–2016) with a linear trend and 95% confidence intervals for English Cocker Spaniels (*n* = *10,313)* among all dogs (*n* = *336,865)* under UK primary veterinary care from January 1^st^, 2016 to December 31^st^, 2016 at practices participating in the VetCompass™ Programme

The study population of 336,865 dogs from 438 clinics in VetCompass™ under veterinary care during 2016 included 10,313 (3.06%) ECS. Of the ECS with information available, 4,878 (47.41%) were females and 4,512 (43.86%) were neutered (Table 1). Proportional neuter status did not differ between females and males (44.30% and 43.46%, respectively, chi-square test: *P* = 0.400). The overall median age was 4.57 years (inter-quartile range (IQR) 2.25–8.01, range 0.18–18.59). Annual proportional birth rates showed relatively stable breed popularity during 2005–2016, ranging between 2.97–3.51% of all births annually (Fig. 1).

**Table 1:** Demography of 10,313 English Cocker Spaniels under primary veterinary care at practices participating in the VetCompass™ Programme in the UK from January 1^st^ to December 31^st^, 2016

Variable	Category	Female (%)^a^	Male (%)^a^	Total (%)^a^
Neuter		2161 (44.30)	2351 (43.46)	4512 (43.85)
Bodyweight (kg)	< 12	810 (21.74)	208 (4.96)	1018 (12.84)
	12 to < 14	1138 (30.55)	682 (16.26)	1821 (22.97)
	14 to < 16	907 (24.35)	1104 (26.32)	2011 (25.37)
	16 to < 18	512 (13.74)	1002 (23.89)	1518 (19.15)
	18 to < 20	226 (6.07)	641 (15.28)	868 (10.95)
	> 20	132 (3.54)	557 (13.28)	691 (8.71)
Age (years)	< 1.0	141 (2.95)	170 (3.18)	312 (3.08)
	1.0 to < 2.0	838 (17.55)	1095 (20.51)	1935 (19.12)
	2.0 to < 3.0	522 (10.93)	686 (12.85)	1208 (11.93)
	3.0 to < 5.0	939 (19.66)	1009 (18.90)	1949 (19.25)
	5.0 to < 7.0	750 (15.71)	814 (15.25)	1567 (15.48)
	7.0 to < 9.0	596 (12.48)	611 (11.44)	1209 (11.94)
	9.0 to < 11.0	485 (10.16)	492 (9.22)	980 (9.68)
	11.0 to < 13.0	336 (7.04)	299 (5.60)	635 (6.27)
	≥ 13.0	168 (3.52)	163 (3.05)	331 (3.27)

^a^Counts cover dogs with available data

**Fig. 2:**
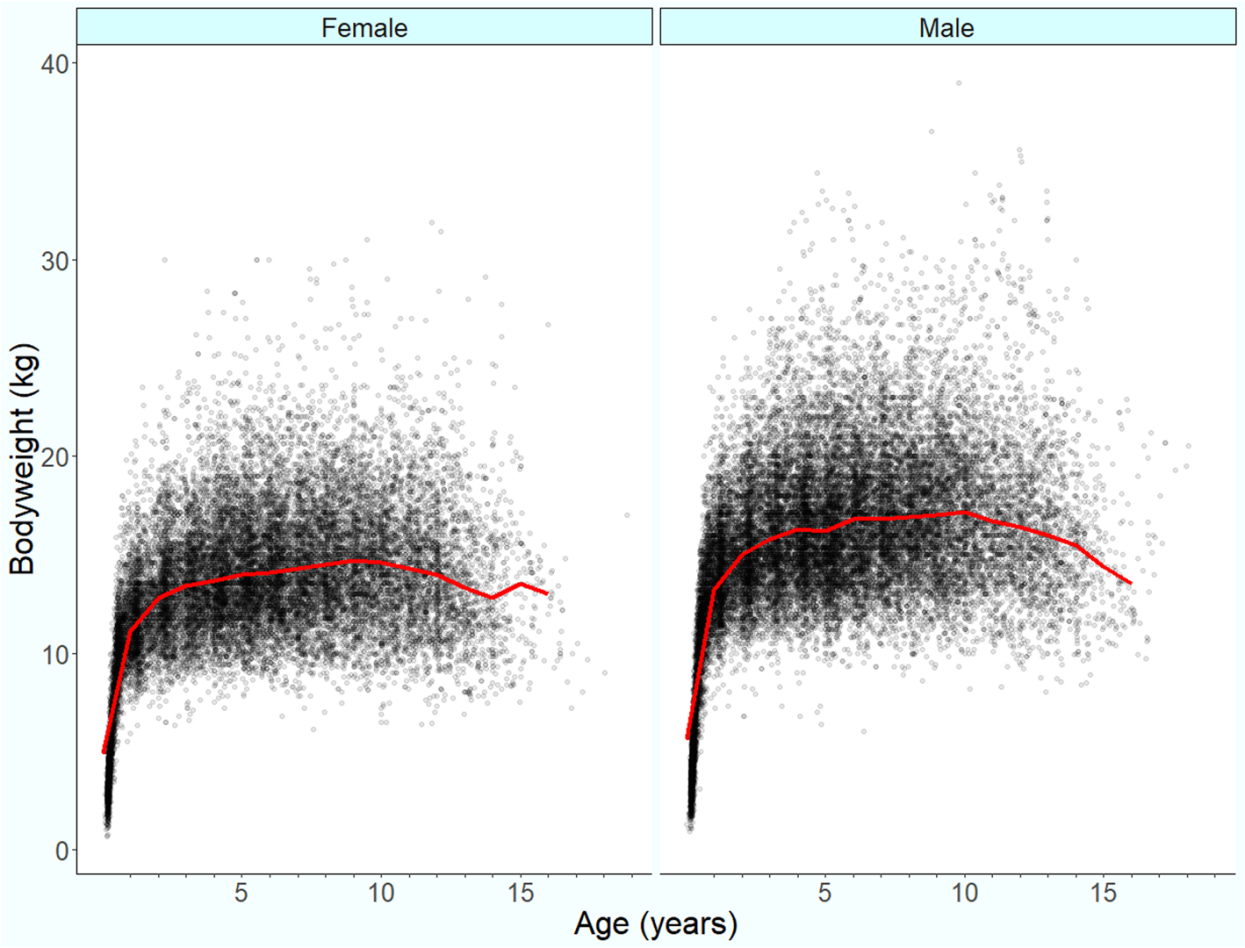
Adult bodyweight at different life stages with a cross medians line plot for female (*n* = 4,069) and male (*n* = 4,698) English Cocker Spaniels under UK primary veterinary care from January 1^st^_,_ 2016 to December 31^st^, 2016 at practices participating in the VetCompass™ Programme

The median adult bodyweight was 15.05 kg (IQR 13.12–17.35, range 6.50–34.13). Males (16.14, IQR 14.25–18.35, range 6.80–34.13) were heavier than females (13.80, IQR 12.21–15.76, range 6.50–30.22) (Mann–Whitney U test: *P* < 0.001). The median bodyweight across all ages was also higher in males (15.18, IQR 13.00–17.58, range 1.78–34.13) than in females (13.10, IQR 11.09–15.20, range 1.05–30.22) (Mann–Whitney U test: *P* < 0.001). Bodyweight curves based on 27,685 bodyweight values in 4,698 males and 23,169 bodyweight values in 4,069 females showed that the ECS grow rapidly during their first year and continue to gain weight until around four years of age (Fig. 2).

The proportional completeness for each variable was sex 99.76%, neuter 99.76%, mean adult bodyweight 76.86%, and age 98.19%.

### Disorder prevalence

The EPRs from a random sample of ECS (2,318/10,313, 22.48%) were manually examined to extract all information on disorders recorded during 2016. Of these, 1,638 (70.66%) dogs had at least one disorder recorded during 2016, while the others received only prophylactic care or no active veterinary care during 2016. During 2016, there were 3,808 unique disorder events reported, and the median annual disorder count was 1 (IQR 0–2, range 0–15) disorder per ECS. The medial annual disorder count was not significantly different between females (1, IQR 0–3, range 0–13) and males (1, IQR 0–2, range 0–15) (Mann–Whitney U test, *P* = 0.464).

There were 342 fine-level disorders reported during 2016, of which the most common were periodontal disease (*n* = 486, prevalence 20.97%, 95% CI: 19.31–22.62), otitis externa (*n* = 234, 10.09%, 95% CI: 8.87–11.32), obesity (*n* = 229, 9.88%, 95% CI: 8.66–11.09), anal sac impaction (*n* = 187, 8.07%, 95% CI: 6.96–9.18), and diarrhoea (*n* = 113, 4.87%, 95% CI: 4.00–5.75) (Table 2). Among the 31 most common fine-level disorders, females had a higher probability of periodontal disease, obesity, and mammary mass lesions, while males had a higher probability of aggression (two-proportion z-test: *P* < 0.05). The median age of dogs with the 31 most common fine-level diagnoses varied from 1.88 years for postoperative wound complications to 11.57 years for cataract (Table 2).

**Table 2:** Prevalence of the most common disorders at fine-level diagnostic precision in English Cocker Spaniels (*n* = 2,318) under primary veterinary care at practices participating in the VetCompass™ Programme in the UK from January 1^st^ to December 31^st^, 2016

Fine-level disorder	Count	Prevalence (%)^a^	Female prevalence (%)	Male prevalence (%)	*p*-value^b^	Median age (years) of dogs with the condition
Periodontal disease	486	20.97 (19.31–22.62)	23.18	19.15	0.020	7.10
Otitis externa	234	10.09 (8.87–11.32)	9.77	10.36	0.689	5.75
Obesity	229	9.88 (8.66–11.09)	12.55	7.69	< 0.001	5.69
Anal sac impaction	187	8.07 (6.96–9.18)	8.72	7.54	0.336	4.50
Diarrhoea	113	4.87 (4.00–5.75	4.69	5.02	0.787	2.92
Aggression	93	4.01 (3.21–4.81)	2.87	4.95	0.015	4.17
Cutaneous mass lesion	73	3.15 (2.44–3.86)	3.16	3.14	1.000	8.95
Lameness	68	2.93 (2.25–3.62)	2.68	3.14	0.599	5.56
Conjunctivitis	65	2.80 (2.13–3.48)	2.87	2.75	0.955	4.75
Postoperative wound complication	65	2.80 (2.13–3.48)	1.92	3.53	0.026	1.88
Vomiting	60	2.59 (1.94–3.23)	2.01	3.06	0.146	3.62
Foreign body	58	2.50 (1.87–3.14)	1.82	3.06	0.077	3.78
Overgrown nail(s)	58	2.50 (1.87–3.14)	2.30	2.67	0.665	3.58
Postoperative complication	57	2.46 (1.83–3.09)	2.01	2.83	0.261	2.96
Aural discharge	47	2.03 (1.45–2.60)	1.53	2.43	0.167	4.29
Ear disorder	43	1.86 (1.31–2.40)	2.30	1.49	0.201	6.91
Flea infestation	42	1.81 (1.27–2.35)	1.82	1.81	1.000	3.71
Hair coat disorder	41	1.77 (1.23–2.31)	1.92	1.65	0.743	3.72
Behavioural disorder	38	1.64 (1.12–2.16)	1.25	1.96	0.235	2.92
Keratoconjunctivitis sicca	38	1.64 (1.12–2.16)	2.01	1.33	0.266	9.46
Subcutaneous mass lesion	36	1.55 (1.05–2.06)	1.63	1.49	0.923	9.19
Cardiac murmur	35	1.51 (1.01–2.01)	1.82	1.26	0.349	8.74
Lipoma	35	1.51 (1.01–2.01)	1.92	1.18	0.201	9.43
Ocular discharge	31	1.34 (0.87–1.80)	1.44	1.26	0.845	7.54
Papilloma	31	1.34 (0.87–1.80)	1.82	0.94	0.099	8.35
Dental disorder	30	1.29 (0.83–1.75)	1.53	1.10	0.463	5.56
Musculoskeletal pain	30	1.29 (0.83–1.75)	1.44	1.18	1.000	8.68
Mammary mass lesion	29	1.25 (0.80–1.70)	2.78	0.00	< 0.001	9.51
Pyoderma	29	1.25 (0.80–1.70)	1.05	1.41	0.558	4.14
Cataract	28	1.21 (0.76–1.65)	1.44	1.02	0.470	11.57
Halitosis	28	1.21 (0.76–1.65)	0.86	1.49	0.235	5.43

^a^Shown as % (95% confidence interval)

^b^Comparing the prevalence in males and females with two-proportion z-test

Information on coat colour was available for 1,483 (63.98%) of the 2,318 dogs. Of these, the most commonly reported colours were black (*n* = 318, 21.04%), liver (*n* = 208, 13.77%), golden (*n* = 207, 13.70%), black & white (*n* = 172, 11.38%), blue roan (*n* = 153, 10.12%), liver & white (*n* = 54, 3.57%), and red (*n* = 41, 2.71%). Of the dogs with recorded colour, 872 (58.80%) were solid-coloured, 573 (38.64%) bi-coloured, and 38 (2.56%) tri-coloured. An association between coat colour and aggression was identified (chi-square test*: P* = 0.024), and pairwise comparisons revealed that solid-coloured dogs had a higher prevalence of aggression compared to bi-coloured (prevalence: 7.00%, 95% CI: 5.30–8.69% and 3.66%, 95% CI: 2.13–5.20%, respectively, two-proportion z-test: *P* = 0.010). Further, the prevalence of aggression varied significantly in the four most common solid colours (black, liver, golden, red) (chi-square test: *P* = 0.017). The coat colour associated with the highest prevalence of aggression was golden (prevalence: 12.08%, 95% CI: 7.64–16.52), followed by red (prevalence: 6.52%, 95% CI: 2.24–17.50%), black (prevalence: 6.29%, 95% CI: 3.62–8.96), and liver (prevalence: 4.33%, 95% CI: 2.29–8.02%).

There were 53 group-level disorders reported during 2016, of which the most common were dental disorders (*n* = 518*,* prevalence = 22.35%, 95% CI: 20.65–24.04), aural disorders (*n* = 319*,* 13.76%, 95% CI: 12.36–15.16), ophthalmic disorders (*n* = 238*,* 10.27%, 95% CI: 9.03–11.50), obesity (*n* = 229*,* 9.88%, 95% CI: 8.66–11.09), and cutaneous disorders (*n* = 228*,* 9.84%, 95% CI: 8.62–11.05) (Table 3). Among the 20 most common group-level disorders, females had a higher probability of dental disorders, obesity, masses, and urinary tract disorders, while males had a higher probability of behavioural disorders (*P* < 0.05, two-proportion z-test). The median age of dogs with the most common group-level disorders ranged from 2.57 years for complications associated with clinical care to 9.99 years for cardiac-related disorders (Table 3).

**Table 3:** Prevalence of the most common disorders at group-level diagnostic precision in English Cocker Spaniels (*n* = 2,318) under primary veterinary care at practices participating in the VetCompass™ Programme in the UK from January 1^st^ to December 31^st^, 2016

Group-level disorder	Count	Prevalence (%)^a^	Female prevalence (%)	Male prevalence (%)	*p*-value^b^	Median age (years) of dogs with the condition
Dental	518	22.35 (20.65–24.04)	24.62	20.49	0.020	6.88
Aural	319	13.76 (12.36–15.16)	13.41	14.05	0.701	5.72
Ophthalmologic	238	10.27 (9.03–11.50)	10.44	10.13	0.857	7.68
Obesity	229	9.88 (8.66–11.09)	12.55	7.69	< 0.001	5.69
Cutaneous	228	9.84 (8.62–11.05)	8.81	10.68	0.153	5.35
Enteropathy	223	9.62 (8.42–10.82)	8.62	10.44	0.159	3.46
Anal sac	206	8.89 (7.73–10.05)	9.39	8.48	0.489	4.73
Mass-associated	190	8.20 (7.08–9.31)	10.25	6.51	0.001	8.56
Musculoskeletal	171	7.38 (6.31–8.44)	7.18	7.54	0.809	6.75
Behavioural	142	6.13 (5.15–7.10)	4.60	7.38	0.007	3.72
Complication associated with clinical care	116	5.00 (4.12–5.89)	4.02	5.81	0.062	2.57
Neoplasia	105	4.53 (3.68–5.38)	5.27	3.92	0.148	9.13
Claw/nail	85	3.67 (2.90–4.43)	2.87	4.32	0.084	4.37
Parasitic	79	3.41 (2.67–4.15)	2.87	3.85	0.242	3.04
Traumatic injury	77	3.32 (2.59–4.05)	2.87	3.69	0.330	4.44
Upper respiratory tract	70	3.02 (2.32–3.72)	2.59	3.38	0.326	5.20
Foreign body	58	2.50 (1.87–3.14)	1.82	3.06	0.077	3.78
Cardiac	53	2.29 (1.68–2.89)	2.68	1.96	0.311	9.99
Female reproductive (females only)	50	2.16 (1.57–2.75)	4.79	-	-	3.72
Urinary	44	1.90 (1.34–2.45)	2.78	1.18	0.008	6.79

^a^Shown as % (95% confidence interval)

^b^Comparing the prevalence in males and females with two-proportion z-test

**Fig. 3:**
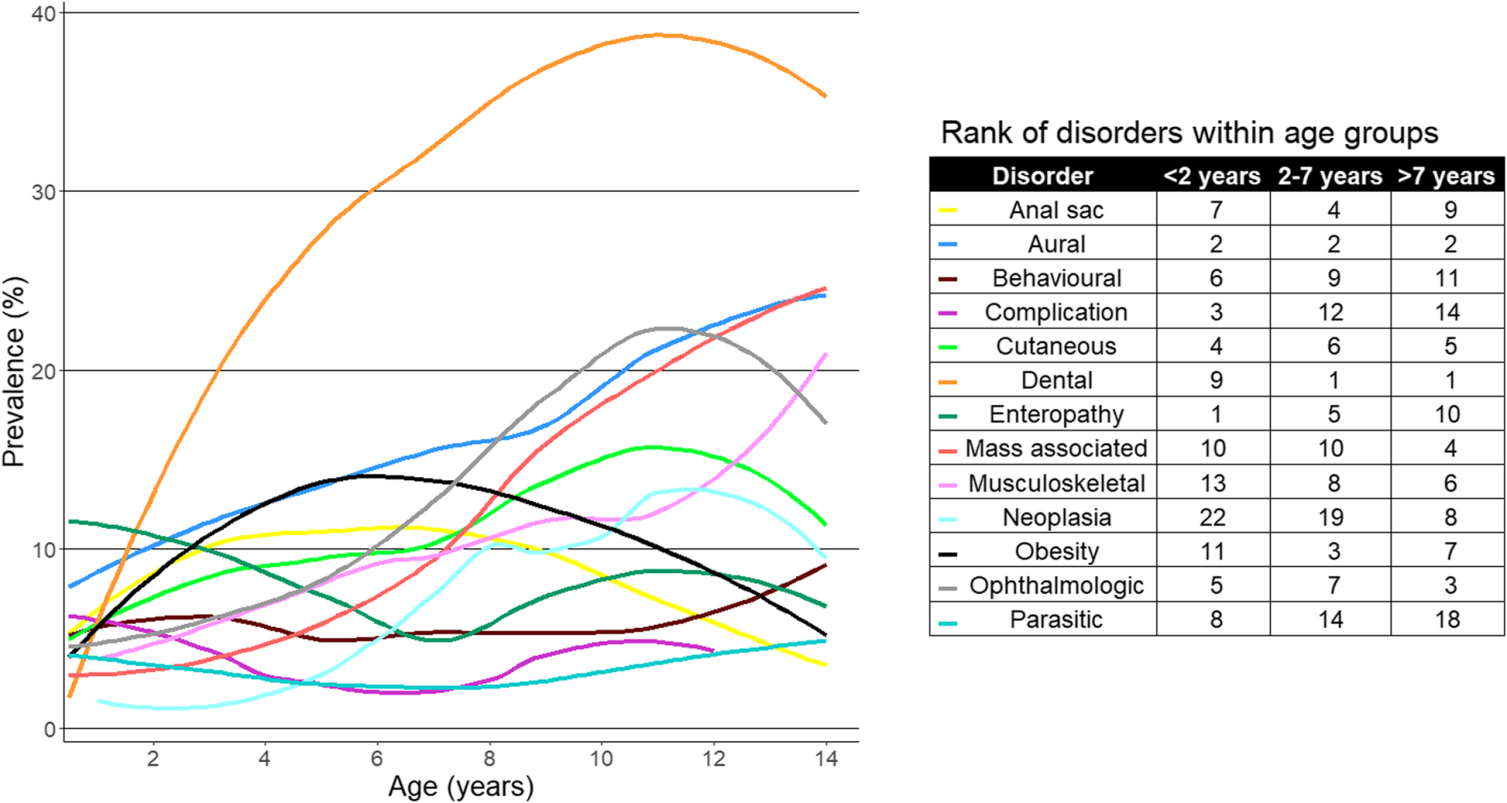
Prevalence of the 10 most common group-level disorders within each of three age bands (< 2 years *n* = *635*, 2–7 years *n* = *1,432*, > 7 years *n* = *1,530*) in English Cocker Spaniels under primary veterinary care at UK practices participating in the VetCompass™ Programme from January 1^st^ to December 31.^st^, 2016

The prevalence of the top 10 most common group-level disorders in three age bands: < 2 years, 2–7 years, and > 7 years is presented in Fig. 3. There were 635 dogs aged under 2 years, 1,432 dogs aged from 2 to 7 years, and 1,530 dogs aged over 7 years. The prevalence of all disorders except behavioural disorders (12/13, 92.31%) varied significantly between the age groups (chi-square test, *P* < 0.05).

### Mortality

In total, 116 of the random sample of 2,318 ECS died during the study period. The median age at death was 11.44 years (IQR 9.46–13.47, range 0.01–17.03). Females showed some evidence of living longer than males (median longevity 12.01 years, IQR 10.25–14.06, range 0.01–16.38, *n* = 59, and 11.00 years, IQR 9.02–13.91, range 1.27–17.03, *n* = 57, respectively) (Mann–Whitney U test, *P* = 0.052). Of 111 (95.69%) deaths with a recorded method of death, 107 (96.40%) were euthanised and 4 (3.60%) died unassisted.

The cause of death was reported for 108 (93.10%) deaths. The most common causes of death at group level precision were neoplasia (*n* = 10, 9.26%, 95% CI: 3.79–14.73), mass-associated disorders (*n* = 9, 8.33%, 95% CI: 4.45–15.08), and collapse (*n* = *8*, 7.41%, 95% CI: 3.80–13.94) (Table 4).

**Table 4:** Mortality in 108 English Cocker Spaniels with a recorded cause of death under primary veterinary care at UK practices participating in the VetCompass™ Programme from January 1^st^ to December 31^st^, 2016

Group-level disorder	Count	Percent (95% CI^a^)
Neoplasia	10	9.26 (3.79–14.73)
Mass-associated disorder	9	8.33 (4.45–15.08)
Collapse	8	7.41 (3.80–13.94)
Brain disorder	7	6.48 (3.17–12.78)
Heart disorder	7	6.48 (3.17–12.78)
Poor quality of life	7	6.48 (3.17–12.78)
Behavioural disorder	6	5.56 (2.57–11.59)
Renal disorder	6	5.56 (2.57–1.59)
Lower respiratory tract disorder	5	4.63 (1.99–10.38)
Abdominal disorder	4	3.70 (1.45–9.14)
Appetite-associated disorder	4	3.70 (1.45- 9.14)
Enteropathy	4	3.70 (1.45–9.14)
Hepatopathy	4	3.70 (1.45–9.14)
Musculoskeletal disorder	4	3.70 (1.45—9.14)
Spinal cord disorder	3	2.78 (0.95—7.85)
Other	20	18.52 (11.19–25.84)

Separate categories are presented for group-level disorders with ≥ 3 individuals

^a^*CI* confidence interval

## Discussion

This study reports on ECS demographics, disorders, and causes of death during 2016, based on anonymised clinical data from primary-care veterinary practices in the UK. The most prevalent fine-level disorders included periodontal disease, otitis externa, obesity, anal sac impaction, diarrhoea, and aggression, while the most prevalent group-level disorders were dental, aural, ophthalmic, obesity, and cutaneous conditions. Analysis of annual proportional birth rates revealed a relatively stable popularity for the ECS breed in the UK from 2005–2016, ranging between 2.97–3.51% of all births annually. The most common causes of death at group-level precision were neoplastic disease, mass-associated disorders, and collapse.

Periodontal disease was the most prevalent fine-level disorder, affecting 20.97% of the ECS. This value is slightly higher than previously reported prevalence of periodontal disease from studies using a similar methodology in other breeds of comparable size (17.6% in Border Terriers [39], 15.7% in West Highland White Terriers [40], 17.4% in Miniature Schnauzers [41]), and is also higher than the general prevalence of periodontal disease in dogs attending primary-care practices in the UK during the same year (12.5%) [42]. Both the ECS and Spaniel breeds in general have been previously identified as predisposed to periodontal disease [42]. The rationale for this predisposition is not clear, but both breed-related and individual differences in the response to plaque on the tooth surface have been suggested [43]. Plaque contains bacteria, and build-up on the tooth surface causes and is often associated with the severity of periodontitis [43, 44]. Further, suspected xerostomia (dry mouth due to decreased or absent saliva flow) has been reported in ECS, which potentially could increase the risk of periodontal disease [45]. However, xerostomia was not commonly diagnosed in the current study. The prevalence of dental disorders increased with increasing age, which correlates well with previous studies that reported increasing age as a risk factor for periodontal disease [42, 43, 46]. Dental disorders are reported to have the highest welfare impact of common disorders in dogs overall, driven mainly by the high prevalence and long duration [25]. Thus, the finding of a high prevalence of periodontal disease in ECS highlights the importance of oral examination in this breed presenting at veterinary practices, especially in older dogs as the prevalence increased with age, followed by a discussion with the dog owner about the dog's risk of periodontal disease and devising a prevention and treatment plan [43]. It should be noted that, although high, the prevalence of periodontal disease in the current study is still likely underestimated, as thorough examination during general anaesthesia is needed to evaluate the full extent of periodontal disease [43]. Further, it cannot be assumed that every veterinary visit where periodontal disease was identified resulted in this description being formally recorded in the EPR.

Otitis externa was the second most common fine-level disorder in the current study, with a prevalence of 10.09%, and also showing increasing prevalence with age. This prevalence in ECS is higher than the 7.3% prevalence for otitis externa in dogs overall attending primary-care practices in the UK during the same year [47] but lower than that reported in a general population of dogs attending a veterinary clinic in Nova Scotia (15.9%) although the latter study used a different methodology to the current study [48]. It is comparable to the prevalence of otitis externa in West Highland White Terriers (10.6%) [40] but higher than in the Border Terrier (6.71%) [39] and Miniature Schnauzers (5.0%) [41] in studies using similar methodology. English Cocker Spaniels and other Spaniel breeds such as the Brittany Spaniel and the Spaniel designer crosses such as Cockapoo and Cavapoo are reported as predisposed to otitis externa [47, 49]. Further, ECS were over-represented in a study of severe otitis externa requiring total ear canal ablation with lateral bulla osteotomy [50]. The causes of otitis externa can be classified according to the PPPS system: primary, predisposing, perpetuating, and secondary causes [51, 52]. Allergic skin disease, endocrinopathies, keratinisation disorders, and immune-mediated disorders are all primary factors that can initiate the inflammatory process, which might be complicated by a secondary yeast or bacterial infection. The predisposing factors contribute to the development of otitis externa, and include humidity, ear carriage, and pinna formation, while the perpetuating factors, such as proliferative changes or stenosis of the ear canal, can prevent resolution and result in relapse [47, 51, 52]. Allergic skin disease could contribute to the elevated prevalence of otitis externa in the current study, as up to 63.6% of dogs with atopic dermatitis and 69% of dogs with cutaneous manifestation of adverse food reactions are affected by otitis externa [10, 49, 53–56]. A breed predisposition in ECS to food allergy and/or atopic dermatitis has been reported in some studies [54, 56, 57], but not in others [54, 58, 59]. Moreover, allergic skin disorder was not included among the top 31 fine-level disorders in the current study, and decreased risk of chronic itching, dermatitis, and allergic skin disorder in ECS compared to other pedigree breeds registered in the UK Kennel Club has been reported [60]. Another primary factor that could contribute to the increased prevalence of otitis externa within ECS is vitamin A-responsive dermatosis, a keratinisation-related disorder associated with ceruminous otitis [61]. Distinct differences in pathologic characteristic of the horizontal ear canal has been reported in ECS compared to other breeds, with a ceruminous tissue response pattern [50]. Finally, the pendulous ear shape of the breed is likely a predisposing factor, as a pendulous ear shape has been reported to increase the risk of otitis externa in several studies [47–49, 62, 63].

The UK Kennel Club describes the ECS as "the merry cocker" with a wagging tail and a happy temperament [1]. This contrasts with the relatively high prevalence of aggression identified in the current study. Aggression was the sixth most common diagnosis at fine-level precision, with a prevalence of 4.01% which is almost twice the 2.24% prevalence of aggression reported in the general population of dogs attending primary-care practices in the UK using a similar methodology to the current study [18]. The prevalence of aggression in ECS is comparable to the 4.2% reported in Chihuahuas [64] but higher than reported in studies of Cavalier King Charles Spaniels [65], Pugs [66], Border Terriers [39], and Miniature Schnauzers [41], where aggression was not even included on the list of the most prevalent disorders. Further, behaviour-related problems were the seventh most common reason for euthanasia in the current study, and a previous study reported that the ECS was the breed with the highest odds of mortality due to undesirable behaviour in dogs under the age of three years [67]. "Rage syndrome" with aggression displayed "suddenly and without apparent reason" has been historically suggested to exist within the breed [21], and ECS are over-represented in studies evaluating canine aggression [16, 17]. Serious, repeated growling and skin-penetrating bites have been reported as the main behaviour-related problems of aggression in ECS [68], and the breed was classified as "very aggressive" in a study of veterinarians' opinions regarding aggression in different dog breeds [69]. Male dogs had a higher prevalence of aggression than females in the current study, in line with previous research [16, 18, 19]. In addition, a significantly higher prevalence of aggression in solid-coloured ECS was found, with the highest prevalence in golden-coloured dogs (12.1%). Previous research has reported that solid-coloured ECS are more likely to show signs of aggression than bi-coloured or tri-coloured, and that golden and red-coated are more likely to show aggression than black-coated [20–23]. The background to this association is not fully known, although a 20% heritability of dominant behaviour within the breed has been reported [22]. As a popular family pet [1], the relatively high prevalence of aggression within the ECS highlights the importance of focusing good breeding on behavioural as well as physical health. The results suggest that solid-coloured males, especially of golden colour, are predisposed to aggression, and hence, prospective ECS owners who are worried about potential breed-related aggression could opt instead for a female, bi-coloured dog.

The prevalence of the 10 most common disorders varied significantly across the three age groups (< 2 years, 2–7 years, and > 7 years) examined. Some disorders, such as complications associated with clinical care, enteropathy, and parasitic disorders, showed a bimodal pattern with peaks in the younger and oldest age groups but had a lower prevalence in middle-aged dogs. Others, such as neoplasia, cutaneous, musculoskeletal, dental, ophthalmologic, aural, and mass-related disorders had prevalence profiles that increased with age. Although different methods of grouping disorder terms across studies make it challenging to compare disorder prevalence through life with studies done by other research teams, the current results correlate generally well with previous reports. Studies based on Swedish insurance data reported an age-related increasing risk of neoplastic disorders, and a trend of age-related increasing risk for ophthalmologic disorders, disorders affecting the integumentary system, and joint-related disorders [70, 71]. In Japanese insurance data, the prevalence of neuromuscular, ophthalmologic, dental, dermatologic, and neoplastic disorders increased with age, while the prevalence of digestive disorders peaked in young and old dogs similar to the findings for enteropathy in the current study [72].

The UK Kennel Club lists pancreatitis, IMHA, and thyroid conditions as current key priorities for ECS [25]. An increased risk of acute and chronic pancreatitis in ECS has been reported in some previous publications [13, 60, 73, 74] but not in others [75, 76]. Chronic pancreatitis is associated with non-specific clinical signs such as inappetence, diarrhoea, vomiting, and lethargy, which may be low-grade and intermittent [76, 77]. The condition has been suggested to be a part of immune-mediated multi-organ disease in ECS, and the affected dogs might present with concurrent conditions such as keratoconjunctivitis sicca (KCS), glomerulonephritis, anal sac disease, xerostomia (with recurrent dental treatments), atopy, hypothyroidism, and IMHA [45, 77, 78]. Pancreatitis was not among the top 31 fine-level disorders in the current study but anal sac impaction, diarrhoea, KCS, and vomiting were, and it is possible that some dogs with these disorders had concurrent, undiagnosed chronic pancreatitis. Increased odds of both IMHA and thyroid conditions have been reported in the ECS [11, 12, 79–82], although none of these conditions were among the top disorders in this study which might reflect an actual low prevalence or under-diagnosing of the conditions. For example, obesity is a common clinical sign in dogs with hypothyroidism, which generally debut in middle-aged dogs in which obesity peaked in the current study [79, 83, 84]. Thus, undiagnosed hypothyroidism might have contributed to the peak of obesity in middle-aged dogs. Consideration needs to be given to the combined effects of prevalence, severity, and duration of a disorder to assess the welfare impact and there needs to be an understanding that a disease predisposition does not necessarily imply a high disease prevalence or high overall welfare impact [25, 26]. In this study, there were other more prevalent disorders than pancreatitis, IMHA, and thyroid conditions, which also should be identified as priorities for breed health [25]. Although pancreatitis, IMHA, and thyroid conditions may show predisposition in ECS and substantially impact the welfare of the individual affected animals, these conditions were not commonly diagnosed in this population of ECS.

The median adult bodyweight in our cohort of both pedigree and non-pedigree ECS in the UK was 15.0 kg, which is slightly higher than the 13–14.5 kg bodyweight span stated within the UK breed standard for pedigree ECS [1]. Body score condition (BCS) data were not extracted in the current study, so it cannot be evaluated whether the higher bodyweight of the wider general population reflects a genuinely larger type of dog in the non-pedigree component although it could reflect some level of overweight/obesity in the wider population as well [85]. The ECS has previously been identified at high risk of obesity [85, 86], and obesity was the third most common fine-level disorder in the current study with a prevalence of 9.88%. This is higher than in the general population of dogs attending primary veterinary care in the UK during the same period (7.1%) [85], but far below the prevalence of obesity previously reported in ECS attending veterinary practices in China (69.4%) and in the US (43.5%) [86, 87]. However, the latter studies only included dogs with recorded BCS while the current study included all dogs, regardless of reporting of overweight, obesity, or BCS. Under-reporting of obesity in patient records is reportedly common [88, 89], and may have affected the current study also. The prevalence of obesity peaked in middle-aged dogs, which is in concordance with previous research [85–87, 90]. The decreasing prevalence in older dogs could be due to chronic disease [90], although greater longevity in leaner dogs could contribute as decreased life expectancy has been reported in overweight dogs [91, 92]. Obesity has been called an epidemic for pets and there are calls to formally recognise obesity as a disease in the Global Pet Obesity Initiative Position Statement [93, 94]. The condition is associated with an increased risk of several disorders, such as cruciate ligament rupture and osteoarthritis [86, 95, 96]. Obesity is a priority area for health-related welfare improvement and reduced quality of life has been reported in obese dogs [97, 98]. Based on the results from the current study, there is a strong argument that veterinarians should routinely assess and record the BCS of ECS to identify overweight individuals and inform ECS owners about the potential health risks associated with obesity, which hopefully could result in a decreased prevalence of obesity and other associated conditions.

The median age at death in ECS was 11.44 years, with some evidence that females lived longer than males (median longevity 12.01 and 11.00 years, respectively). The median longevity in ECS is similar to the longevity of 11.23–12.0 years in dogs of all breeds attending primary-care veterinary practices in the UK [6, 99], and the 10.33–11.15 years reported in UK Kennel Club registered breeds [5, 100]. It also concords with the median/mean longevity of ECS in previous reports (10.75–11.7 years) [5, 6, 99, 100]. The most common causes of death were neoplasia, mass-associated disorders, and collapse. Cancer was similarly reported as the most common reason for death in UK Kennel Club registered ECS, followed by heart failure and aggression [5]. Further, neoplastic disease was reported as the most common pathophysiologic process associated with mortality in ECS in the US using the Veterinary Medical Database during 1984–2004 [101]. Although the current study was under-powered to explore associations between specific types of neoplasia and mortality, ECS are reported as predisposed to the development of neoplasms in general [102, 103], as well as to some specific types including adenocarcinomas, melanocytic tumours, lipoma, and anal sac gland carcinoma [103–105]. In summary, neoplastic disease is reported as the most common cause of death for ECS and is highlighted as an important life-limiting condition in ECS.

The current study used data from primary-care veterinary practices. Other secondary data sources for disease surveillance in dogs include pet insurance databases and referral practice clinical records [27]. A strength of primary-care data compared to insurance data is the opportunity to evaluate conditions that often have limited insurance coverage, such as periodontal disease, obesity, and behaviour-related disorders [106]. Further, the information from the free-text clinical notes in the EPR can be scrutinised when using primary-care data, whereas insurance-based epidemiologic studies generally are limited to just the clinical information given as the reason for the insurance claim [72, 106, 107]. The results of the current study are likely more generalisable to the wider dog population than results from studies based on data from referral practice clinical records since these might be biased towards more complicated cases requiring specialist care [27, 108]. However, some limitations with using primary-care veterinary data in research have been identified in a previous publication [27]. Given that only 77% of the UK dog population is estimated to be registered at veterinary practices, the generalisability of the results from the current study to the unregistered population is unknown [28]. There is a risk of bias in the estimation of the annual proportional birth rates, both due to differential longevity and popularity profiles across breeds and the inclusion of fewer individuals in the earlier years resulting in lower precision of estimates [99]. Further, the mortality data included relatively few individuals, which should be considered when the results are interpreted. There is also a risk of under-reporting of disorders, which likely is higher for conditions other than the main reason for the veterinary examination. It is possible that some of the included dogs attended veterinary practices outside the VetCompass Programme during 2016, resulting in under-reporting of disorders diagnosed at these practices. Some of the reported diagnostic terms were clinical signs, and there is a risk of under-reporting of the specific underlying biomedical diagnoses, such as pancreatitis, hypothyroidism, and atopic dermatitis. In summary, this study reports on disorders diagnosed in ECS at the included veterinary practices, not the true prevalence of disorders within the breed. It should also be highlighted that this study reports the prevalence of disorders, not the incidence. The age reported for each condition represents the age of affected dogs on 31 December 2016 and not the age at first diagnosis of the condition.

The classification of dogs as aggressive or not was based on the information in the patient records, which was not detailed enough to allow for further characterisation or grading of the severity of behaviours related to aggression. Given that the study intended to explore the demography, longevity, and common disorders of ECS, the study included all dogs registered as Cocker Spaniels and ECS but excluded other variants of Cocker Spaniels such as American Cocker Spaniels. However, there is still some risk of misclassification bias for the breed in the study. There is also a risk of misclassification bias for coat colour, which was classified according to the information recorded in the EPR. Finally, the current study reports the prevalence of common conditions affecting the ECS but does not provide information on the duration and severity of the conditions, which are also important to consider when the total welfare impact of a disease is assessed [25].

## Conclusion

This study of over ten thousand ECS under primary veterinary care in the UK reported demographics, disorder data, and mortality. The most prevalent disorders were periodontal disease, otitis externa, and obesity, while the most common causes of death were neoplasia and mass-associated disorders. The prevalence of aggression was higher in males and in solid-coloured dogs, with the highest prevalence in golden-coated dogs. The results can aid veterinary surgeons in giving evidence-based breed-adapted health information to dog owners and support breeding organisations by identifying priorities for ECS health and welfare.

## Data Availability

The dataset supporting the conclusions of this article will be made available in the RVC Research Online repository.

## Abbreviations

BCS: Body condition score
CI: Confidence interval
ECS: English Cocker Spaniel
EPR: Electronic patient record
IMHA: Immune-mediated haemolytic anaemia
IQR: Inter-quartile range
